# A collision tumor of basal cell carcinoma and atypical fibroxanthoma: A case report

**DOI:** 10.1002/ccr3.9250

**Published:** 2024-08-20

**Authors:** Mariko Suzuki‐Ueno, Yoshiaki Fujikawa, Dai Hamaoka, Kaoru Umemura, Takamasa Ohnishi

**Affiliations:** ^1^ Department of Dermatology Nishiwaki Municipal Hospital Nishiwaki Japan; ^2^ Department of Diagnostic Pathology Nishiwaki Municipal Hospital Nishiwaki Japan

**Keywords:** atypical fibroxanthoma, basal cell carcinoma, collision tumor, skin neoplasms

## Abstract

An 83‐year‐old man presented an elevated skin lesion in the left temporal area. The resected specimen was identified between a basal cell carcinoma and an atypical fibroxanthoma. A final diagnosis of basal cell carcinoma and atypical fibroxanthoma was made. This study reports a rare case of a cutaneous collision.

## 1 INTRODUCTION

Collision tumors and skin lesions are neoplastic lesions comprising two or more distinct cell populations separated by well‐defined boundaries. These may comprise two benign tumors, a benign tumor and a malignant tumor, or two malignant tumors.^1^ Basal cell carcinoma (BCC) is a relatively common type of skin carcinoma that originates from the interfollicular epidermis and basal cells of the hair follicle.^2^ It is the most common skin cancer in Japan, with an annual incidence of approximately 3.34 per 100,000 individuals.^3^ In contrast, atypical fibroxanthoma (AFX), which was first reported by Lund and Kraus in 1962,^4^ is a relatively rare, malignant, low‐grade sarcoma, accounts for 0.2% of all skin tumors,^5^ and originates in the dermis, spreading into the subcutaneous tissue. Owing to its histological features, AFX is also considered a superficial type of malignant fibrous histiocytoma.^6^ Both BCC and AFX typically develop in sun‐damaged skin, especially on the head and neck, of older adults. Immunohistochemical analyses have revealed that specimens obtained from patients with BCCs are positive for p40, p63, and CK5/6,^7^, ^8^ whereas those obtained from patients with AFXs are positive for CD10, CD68, and CD163.^9^, ^10^, ^11^

Tumors with both BCC and AFX components are extremely rare. To the best of our knowledge, only three such cases have been previously reported.^12^, ^13^, ^14^ Here, we report a rare case of a cutaneous collision tumor, with co‐occurring BCC and AFX, in an 83‐year‐old man.

## 2 CASE HISTORY/EXAMINATION

**FIGURE 1:**
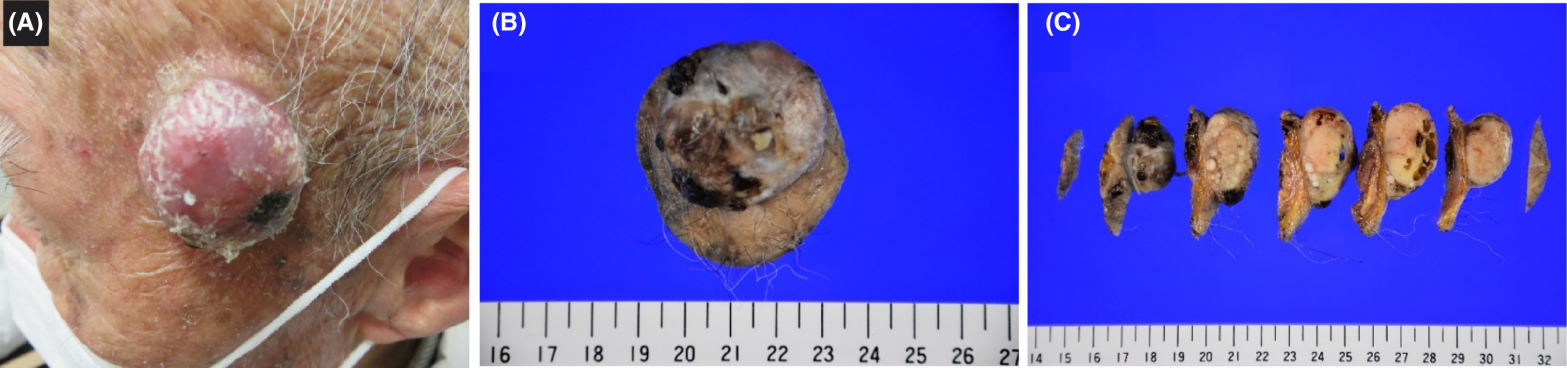
Macroscopic images of the tumor. (A) Clinical photograph. A 30 × 25‐mm, elevated, erythematous skin mass is observed in the left temporal area. (B) Macroscopic image of the resected specimen. (C) The excised specimens are divided into seven sections. The mass is pale and yellowish white, with partially scattered white nodules and black spots reminiscent of bleeding. Multiple small cysts are observed just below the epidermis.

An 83‐year‐old man presented to our dermatology department with a skin lesion in the left temporal area. Seven months before the first visit, he observed a mass in the left temporal area, which was prone to bleeding and gradually grew. Before biopsy, the differential diagnosis was a subcutaneous tumor, which was suspected to be malignant. He had a medical history of hypertension, chronic obstructive pulmonary disease, cerebral infarction, hypothyroidism, chronic gastric inflammation, gastroesophageal reflux disease, and immunoglobulin A nephropathy. He had received radiation therapy for laryngeal cancer. On examination, a dome‐shaped tumor of approximately 30 × 25 mm was identified in the left temporal area (Figure 1A).

## 3 METHODS

### 3.1 Differential diagnosis, investigations, and treatment

A punch biopsy of the skin revealed both BCC and AFX components; the histological findings were similar to those of a malignant fibrous histiocytoma. The tumor was surgically excised under local anesthesia with 10‐mm margins along with a portion of the facial muscles. The resected specimen was sent for histopathological and immunohistochemical investigation.

**FIGURE 2:**
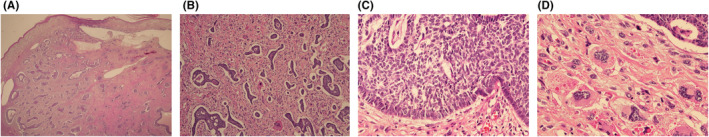
Histological findings of the tumor indicate components of both atypical fibroxanthoma and basal cell carcinoma. (A) Hematoxylin and eosin staining (HE) shows atypical basaloid cells protuberating from the epidermis into the dermis and subcutaneously in the form of cysts. Magnification: ×12.5. (B) HE reveals collision of BCC and AFX. Magnification: ×100. (C) HE shows cleft formation/palisading, typical of basal cell carcinoma. Magnification: ×400. (D) HE shows the proliferation of large monocytes, multinucleated giant cells, and oval‐to‐spindle‐shaped cells in the stroma. Magnification: ×400.

An elastic, firm, elevated lesion measuring 38 × 22 mm was identified (Figure 1B). On the cut surface, the tumor was pale to yellowish white, with partially scattered white nodules and black spots reminiscent of bleeding. The formation of a small cyst was observed just below the epidermis (Figure 1C). Atypical basaloid cells were observed proliferating continuously in a nest‐to‐reticulated manner from the epidermis into the dermis and subcutis. Cleft formation/palisading was observed in some areas. The proliferation of large monocytes and multinucleated giant cells and the flowing proliferation of oval‐to‐spindle‐shaped cells were observed in the stroma. The histological findings were typical of a BCC and an AFX. A low‐magnification image of the tumor (Figure 2A) and three high‐magnification images depict the diagnostic features of BCC and AFX that have collided (Figure 2B), BCC (Figure 2C), and AFX (Figure 2D). In the histopathological evaluation, considering the tumor area ratio, the AFX and BCC components were approximately 70% and 30%, respectively. The surgical margins were clear.

## 4 CONCLUSION AND RESULTS

**FIGURE 3:**
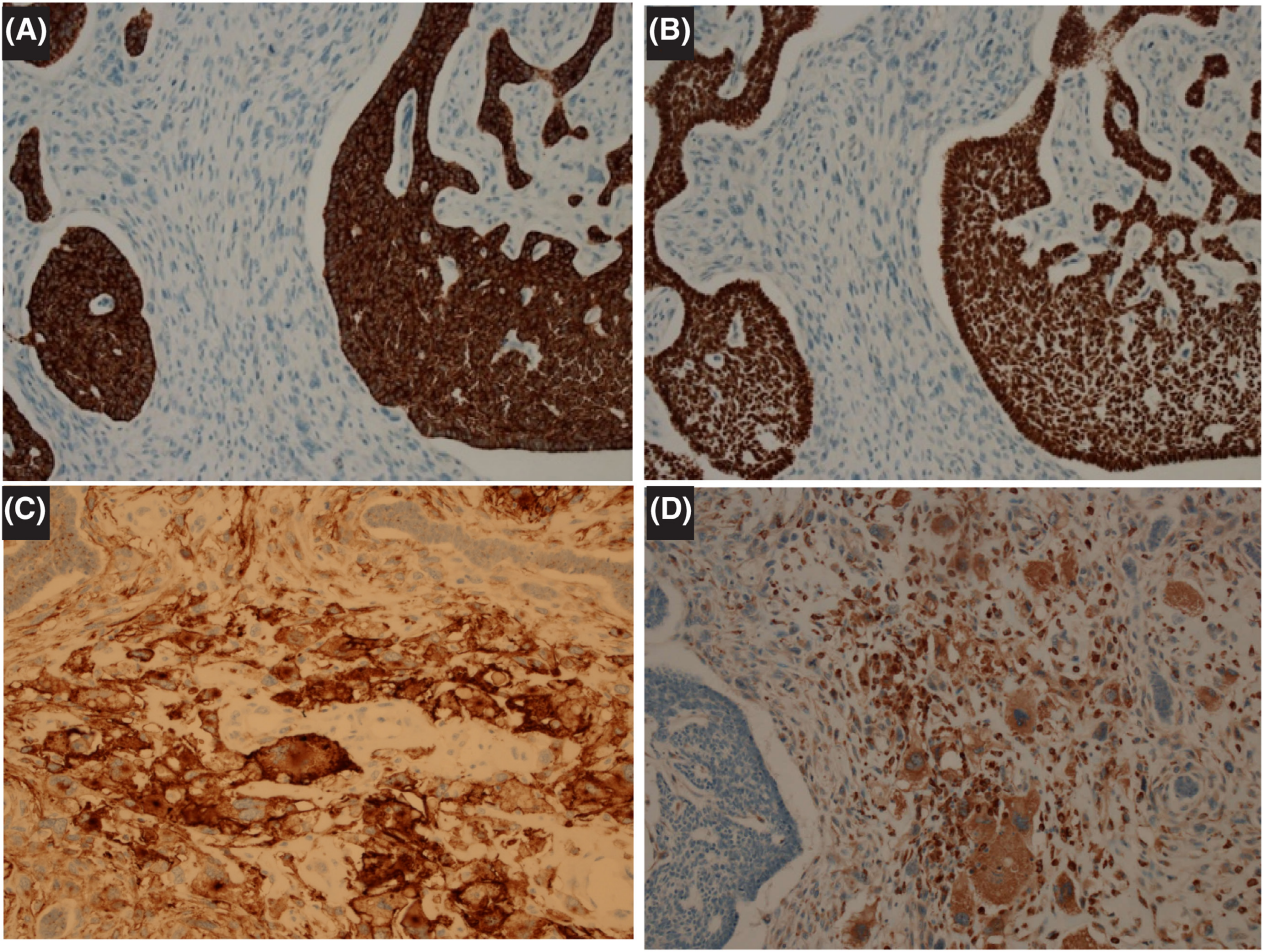
Immunohistochemical staining for basal cell carcinoma. Cells are positive for (A) cytokeratin (CK)5/6 and (B) p40, CK5/6, and p40. Findings from the immunohistochemical staining of the atypical fibroxanthoma. Cells are positive for (C) CD10 and (D) CD68.

The BCC was AE1/AE3 (−), CAM5.2 (+; weakly), cytokeratin (CK) 5/6 (+) (Figure 3A), and p40 (+) (Figure 3B). The AFX was AE1/AE3 (−), CAM5.2 (−), CD10 (+; Figure 3C), CD68 (+; Figure 3D), CD163 (+), α‐smooth muscle actin ([α‐SMA] +; partially), fascin (+), procollagen 1 (+), desmin (−), calponin (−), and S‐100 (−). The Ki‐67 index was 60.5% for BCC and 21.0% for AFX. On the basis of the above‐mentioned histological and immunohistochemical findings, the tumor was diagnosed as having combined components of BCC and AFX.

### 4.1 Outcome and follow‐up

Two years have passed since the excision, with no local recurrence or metastasis.

## 5 DISCUSSION

Collision tumors are difficult to accurately diagnose because of their complex morphology,^1^ necessitating accurate diagnosis of tumor cell morphology and the use of immunohistochemistry. Our report emphasizes the importance of histopathological diagnosis in such cases to accurately identify the tumor type.

Our patient was an older man, whose lesions occurred on the face, which is exposed to ultraviolet light. Three other similar cases^12^, ^13^, ^14^ also involved the face in patients aged >60 years. Histologically, the patient was diagnosed as having a lesion with co‐occurring BCC and AFX. Immunohistochemical findings for BCC revealed that the lesion tested negative for the epithelial markers AE1/AE3 and positive for some CAM5.2. Cutaneous spindle cell squamous carcinomas are often difficult to diagnose in undifferentiated tumor cells and test negative for pan‐cytokeratins (AE1/AE3). However, spindle cell squamous carcinomas have stained positive for CK5/6 in several cases,^8^ and it is worth considering whether they stain negative for pan‐cytokeratin. The tumor cells in our case tested positive for both CK5/6 and p40, indicating that they were derived from the basal cells of the epidermis and hair follicles.^2^ The Ki‐67 index for BCCs ranges between 1%–61%, averaging 12.3%.^15^ Notably, the BCC lesion in our case had a Ki‐67 index of 60.5%, which is considerably high, suggesting increased cell proliferation. The AFX test result was negative for both AE1/AE3 and CAM5.2 and positive for both CD68 and CD163, indicating that it was a nonepithelial tumor with histiocytic traits. CD68 and CD163 positivity have been reported in 79% of AFX cases.^11^ In our case, leiomyosarcoma was ruled out because the tumor tested negative for desmin and calponin. However, the tumor tested partially positive for α‐SMA and positive for fascin. AFXs reportedly show positivity for α‐SMA in approximately 60% of cases^16^ and for fascinas.^17^ Immunohistochemical analyses have revealed that AFXs are positive for both CD10 and procollagen 1.^9^ The tumor in our case tested negative for S‐100, ruling out a nervous system tumor and melanoma. The Ki‐67 index for AFXs ranges between 2.2%–22.2%, averaging 12.2 ± 6.3%.^18^ The AFX in this case had a Ki‐67 index of 21.0%, which is considerably high. Notably, the Ki‐67 indices for both the BCC and AFX components in our case were higher than the average values reported in other cases.^15^, ^18^ Moreover, collision tumors of BCC and AFX have rarely been reported.^12^, ^13^, ^14^ In the context of the development of the soft tissue sarcoma termed AFX, Li‐Fraumeni syndrome does not seem relevant with regard to this patient. Given the older age of onset of AFX and the absence of a family history of cancer, the disease is unlikely to be related to Li‐Fraumeni syndrome.^19^

We report the rare case of a cutaneous collision tumor of co‐occurring BCC and AFX, highlighting the importance of diagnostic histopathology for correctly identifying the tumor type in such cases. Complete tumor remission was achieved with resection. While awaiting additional case reports, further research on this condition is warranted.

## AUTHOR CONTRIBUTIONS

**Mariko Suzuki‐Ueno:** Conceptualization; methodology; resources; visualization; writing – original draft. **Yoshiaki Fujikawa:** Data curation; writing – review and editing. **Dai Hamaoka:** Investigation. **Kaoru Umemura:** Resources. **Takamasa Ohnishi:** Conceptualization; data curation; methodology; project administration; visualization; writing – original draft; writing – review and editing.

## FUNDING INFORMATION

None.

## CONFLICT OF INTEREST STATEMENT

None.

## CONSENT

Written informed consent was obtained from the patient described in this case report for publication of the details of the medical case and any accompanying images. Written informed consent was obtained from the patient to publish this report in accordance with the journal’s patient consent policy

## ETHICAL STATEMENT

Written consent for publication has been obtained from the patient described in this case report.

## Data Availability

Data and material are available from the corresponding author upon request.
